# COPD’s early origins in low-and-middle income countries: what are the implications of a false start?

**DOI:** 10.1038/s41533-019-0117-y

**Published:** 2019-03-05

**Authors:** E. A. Brakema, F. A. van Gemert, R. M. J. J. van der Kleij, S. Salvi, M. Puhan, N. H. Chavannes, the FRESH AIR collaborators, Pham Le An, Marilena Anastasaki, Meerim Akmatalieva, Azamat Akylbekov, Andy Barton, Antonios Bertsias, Pham Duong Uyen Binh, Job F. M. van Boven, Dennis Burges, Lucy Cartwright, Vasiliki E. Chatzea, Liza Cragg, Tran Ngoc Dang, Ilyas Dautov, Berik Emilov, Irene Ferarrio, Ben Hedrick, Le Huynh Thi Cam Hong, Nick Hopkinson, Elvira Isaeva, Rupert Jones, Corina de Jong, Sanne van Kampen, Winceslaus Katagira, Bruce Kirenga, Jesper Kjærgaard, Janwillem Kocks, Le Thi Tuyet Lan, Tran Thanh Duv Linh, Christos Lionis, Kim Xuan Loan, Maamed Mademilov, Andy McEwen, Patrick Musinguzi, Rebecca Nantanda, Grace Ndeezi, Sophia Papadakis, Hilary Pinnock, Jillian Pooler, Charlotte Poot, Maarten J. Postma, Anja Poulsen, Pippa Powell, Nguyen Nhat Quynh, Susanne Reventlow, Dimitra Sifaki-Pistolla, Sally Singh, Talant Sooronbaev, Jaime Correia de Sousa, James Stout, Marianne Stubbe-Østergaard, Aizhamal Tabyshova, Ioanna Tsiligianni, Tran Diep Tuan, James Tumwine, Le Thanh Van, Nguyen Nhu Vinh, Simon Walusimbi, Louise Warren, Sian Williams

**Affiliations:** 1 Department of Public Health and Primary Care, Leiden University Medical Center, Leiden University, Leiden, The Netherlands; 2 Department of General Practice Groningen, Institute for Asthma and COPD, University Medical Center Groningen, University of Groningen, Groningen, The Netherlands; 3 Chest Research Foundation, Pune, India; 4 Epidemiology, Biostatistics and Prevention Institute, University of Zurich, Zurich, Switzerland; 5 University of Medicine and Pharmacy, Ho Chi Minh City, Vietnam; 6 Clinic of Social and Family Medicine, School of Medicine, University of Crete, Heraklion, Greece; 7 Ministry of Health of the Kyrgyz Republic, National Center of Cardiology and Internal Medicine, Bishkek, Kyrgyzstan; 8 Faculty of Medicine and Dentistry, University of Plymouth, Plymouth, UK; 9 University of Groningen, University Medical Center Groningen, Groningen, The Netherlands; 10 Department of Pediatrics, University of Washington School of Medicine, Seattle, WA USA; 11 International Primary Care Respiratory Group, London, UK; 12 European Lung Foundation, Sheffield, UK; 13 Imperial College London, London, UK; 14 Makerere University Lung Institute, College of Health Sciences, Makerere University, Kampala, Uganda; 15 The Research Unit for General Practice and Section of General Practice, Department of Public Health, Copenhagen University, Copenhagen, Denmark; 16 Global Health Unit, The Department of Paediatrics and Adolescent Health, Juliane Marie Center, Copenhagen University Hospital “Rigshospitalet”, Copenhagen, Denmark; 17 National Centre for Smoking Cessation and Training, Dorchester, UK; 18 University of Edinburgh, Usher Institute of Population Health Sciences and Informatics, Edinburgh, UK; 19 Coventry University, Coventry, UK; 20 University of Minho, School of Medicine, Braga, Portugal

The Global Initiative for chronic Obstructive Lung disease (GOLD) guideline of 2018 describes COPD as ‘the result of a complex interplay of long-term cumulative exposure to noxious gases and particles, combined with a variety of host factors including genetics, airway hyper-responsiveness and poor lung growth during childhood’.^1^ Tobacco smoking is traditionally viewed as the main contributing factor to the development of COPD. However, COPD also occurs among non-smokers, especially in low-income and middle-income countries (LMICs).^2,3^ Notably, more than 90% of COPD-related deaths occur in LMICs.^4^ For these countries, other risk factors, such as ambient, occupational and household air pollution play a significant role in the development of COPD.^1,2,5–7^ Does COPD in these settings have a different pathophysiological trajectory compared to COPD in high-income countries, and if so: what does this imply?

In normal lung development, airway branching is completed by the 17th week of gestation, after which airways increase in volume until young adulthood. Alveoli are present at birth and develop further during childhood. Lung volume and airflow continue to increase as the thorax grows, influenced by age, sex, and ethnicity, reaching a peak at young adulthood. Lung function then remains constant for about 10 years (the plateau phase), after which it gradually declines.^8^ In the ‘classic’ COPD patient, the decline in lung function is more rapid than in healthy individuals. However, in a considerable proportion of COPD patients, lung function does not decline rapidly, but reaches a lower plateau phase in early adulthood instead. For these patients, a completely different pathophysiological trajectory seems to lead to the diagnosis of COPD: the decline in lung function follows a normal pattern, yet they seem to have a ‘false start’ by attaining a lower maximum lung function.^8–11^

Several factors can contribute to a lower maximum lung function. There is increasing evidence that different environmental factors (such as tobacco smoke, household air pollution caused by burning solid fuels or kerosene lamps), maternal factors (malnutrition, asthma, diabetes), and gene–environment interactions can impact lung growth. Additionally, childhood factors (low birth weight, prematurity) and other diseases during childhood (lower respiratory tract infections, asthma, tuberculosis, HIV) may all contribute to a decline in lung function.^1–3,5,8,9,11–14^ (Mal)nutrition additionally plays a vital role in lung development.^15^ Unfortunately, many of these factors commonly interrelate and co-occur in LMICs. A low socioeconomic status can furthermore deteriorate the situation, as this is not only independently associated with a lower lung function, but also with other health risks (poor access to healthcare, poor nutrition, low birth weight, exposure to indoor and outdoor pollution, poor living conditions and water supply/sanitation).^16–18^ Overall, populations in LMICs are particularly vulnerable to such risk factors for a lower maximum lung function.^16,18^

Our knowledge about reduced maximum attained lung functions caused by ‘early life disadvantage factors’ in LMICs seems humble compared to the knowledge generated by extensive research on tobacco-related COPD, often conducted in high-income countries.^5,11,13,19^ The question is whether these early life, disadvantageous factors cause different COPD phenotypes with their own pathophysiology, diagnostic approach, rate of lung function decline, response to treatment, and potential reversibility.^3,9,13,19^ The paucity of data indicates differences may exist, and that those with a lower forced expiratory volume in one second in early adulthood are also exposed to an increased prevalence and earlier incidence of other non-communicable diseases and premature death.^5,12,20,21^ Additionally, COPD related to biomass fuel smoke was demonstrated to be commonly present among young, non-smoking women,^6^ whereas ‘classic’ COPD patients are elderly, smoking men. Such difference could be attributed to differences in risk exposure between men and women, yet potential differences in sensitivity to COPD between men and women per phenotype could play an additional role. Furthermore, as rates of lung function decline vary according to the underlying cause of COPD, the approach equally differs. For tobacco or biomass fuel smoke exposure the decline may decelerate once removed from the exposure, whereas a normal decline within patients with a lower plateau phase seems harder to influence.

COPD caused by a false start has several implications. First, COPD caused by early life disadvantage factors should be tackled at much earlier stages. Prevention is key, and must start before birth, continue throughout childhood and then be combined with the ‘regular’ preventive measures targeting an accelerated decline. As early life disadvantage factors are diverse, prevention addressing these factors should be equally diverse. We argue that raising awareness on COPD is therefore crucial. COPD, the third cause of death worldwide, is often completely unknown to local community members, including their healthcare professionals and policy makers.^22^ Local curricula for healthcare professionals (doctors, nurses, midwives) thus need to increase focus on COPD and on its early origin. Moreover, community health workers should be trained on a national scale to facilitate COPD awareness programmes in their communities. Raising awareness among policy makers is also essential. The diverse early life risk factors require COPD interventions to be embedded in a wide, inter-sectoral approach to facilitate a socioeconomic upliftment (targeting amongst others poverty, education, infrastructure, health literacy).

Increased awareness on the early development of COPD and on the existence of COPD in general could enhance early diagnosis and timely action. Increased awareness is particularly important considering the widescale underdiagnosis of COPD. This raises the question how to best diagnose the disease (affordably, easy to learn), and how to do so at an early stage.^6,23^ Although affordable medications to treat COPD in LMICs are rare, non-pharmacological approaches, such as pulmonary rehabilitation and patient education can impact COPD symptoms and disease progression. Particularly in LMICs, these approaches seem cost-effective (if not, cost-saving).^24^

The second implication is related to research. As outlined earlier, more research into a lower maximum lung function and its clinical implications is needed. Furthermore, a false start implies a different perspective on how to study the effect of interventions targeting household air pollution on lung function. For decades, clean cook stove programmes fail to demonstrate consistent positive effects on lung function.^25^ Often this is attributed to flawed implementation of such interventions, or to cross-contamination of air pollution within the community. However, even after successful adoption of clean cook stoves, the lungs in this generation could remain irreversibly damaged. Instead, should we not measure effects on lung function among children and adolescents, or even among the next (unborn) generation?

To conclude, COPD development generally starts early in life due to a complex interplay of disadvantageous factors. Many of these factors particularly occur in LMICs. To address the silent growing epidemic of COPD in LMICs, lung health should be optimised even before birth and in early childhood. Prevention is key, and focus should be on creating large-scale awareness—beyond the healthcare system only, involving all stakeholders. More research is needed to explore the implications of early life disadvantage factors for COPD in LMICs. This should lead to an evidence-based approach for those with a false start—those (at risk of) having a lower maximum lung function.
